# T cell dysregulation reflects disease stage in hepatitis virus and alcohol-related liver disease

**DOI:** 10.1038/s41598-025-18624-4

**Published:** 2025-09-30

**Authors:** Christian Niehaus, Yin-Han Chou, Helena Lickei, Ayesha Lietzau, Roni Souleiman, Benjamin Maasoumy, Heiner Wedemeyer, Christine S. Falk, Anke R. M. Kraft, Markus Cornberg

**Affiliations:** 1 Department of Gastroenterology, Hepatology, Infectious Diseases and Endocrinology, Hannover Medical School, Hannover, Germany; 2 Centre for Individualised Infection Medicine (CiiM), a joint venture between the Helmholtz Centre for Infection Research (HZI) and Hannover Medical School (MHH), Hannover, Germany; 3 TWINCORE, Centre for Experimental and Clinical Infection Research, a joint venture between the Helmholtz Centre for Infection Research (HZI) and the Hannover Medical School, Hannover, Germany; 4 German Center for Infection Research (DZIF), Partner-Site Hannover-Braunschweig, Hannover, Germany; 5 Cluster of Excellence RESIST (EXC 2155), Hannover Medical School, Hannover, Germany; 6 German Center for Infection Research, HepNet Study-House German Liver Foundation, Hannover, Germany; 7 Institute of Transplant Immunology, Hannover Medical School, Hannover, Germany

**Keywords:** Cirrhosis-associated immune dysfunction, Cytokines, Hepatitis virus infection, Liver cirrhosis, Systemic inflammation, T cells, Viral hepatitis, Liver cirrhosis, Alcoholic liver disease, Translational immunology, Chronic inflammation

## Abstract

Liver cirrhosis is characterized by both immunodeficiency and an exaggerated immune response leading to systemic inflammation. In this study, we investigated the role of T cells in different stages of liver disease and therefore analyzed 72 patients stratified into those with and without cirrhosis and compensated and decompensated cirrhosis. Flow cytometry revealed that CD8^+^ T cells, but not CD4^+^ T cells were diminished in patients with decompensated liver cirrhosis and this further resulted in an increased CD4/CD8 T cell ratio in those patients. In addition, the phenotype of CD4^+^ and CD8^+^ T cells shifted towards activated and exhausted effector-memory and terminally differentiated cells in patients with compensated liver cirrhosis, which was parallelled by an impaired functional response upon stimulation with IL-12 + IL-18. Conversely, in patients with decompensated cirrhosis, CD4^+^ and CD8^+^ T cells exhibited heightened proliferative activity and showed a relative loss of activation and exhaustion marker expression compared with compensated cirrhosis. Furthermore, investigation of 64 soluble immune mediators revealed an inflammatory cytokine milieu in patients with decompensated liver cirrhosis. Taken together, these results indicate an association between phenotypic and functional changes in the T cell compartment and the different stages of liver disease.

**Supplementary Information:**

The online version contains supplementary material available at 10.1038/s41598-025-18624-4.

## Introduction

Immune activation represents a critical hallmark in the progression of liver disease, particularly in the context of chronic conditions that lead to fibrosis and, subsequently, liver cirrhosis^1–3^. The disease pattern of liver cirrhosis is characterized by an asymptomatic compensated phase followed by a decompensated phase due to increasing portal pressure and worsening liver function subsequently leading to complications, as evidenced by the development of apparent clinical signs, the most frequent of which are ascites, hepatic encephalopathy, esophageal varices bleeding, and the occurrence of icterus^4–6^.

The progression of liver disease to liver cirrhosis is not only characterized by hepatocyte damage driving fibrogenesis through hepatic stellate activation; it is also marked by increasing alterations in the immune system, which is termed cirrhosis-associated immune dysfunction (CAID) syndrome^1,7^. CAID compromises the distinct spectrum of immune alterations associated with the course of end-stage liver disease. On the one hand, it involves systemic immune dysfunction. On the other hand, it comprises a state of systemic hyperinflammation, driven by persistent immune cell activation and increased levels of pro-inflammatory cytokines^1,8^. In more detail, CD4^+^ and CD8^+^ T cells play pivotal roles in both protective and pathogenic mechanisms. Indeed, CD8^+^ T cells are crucial for antiviral responses and tumor surveillance in the liver^9–12^. However, in chronic liver diseases, persistent antigen stimulation results in sustained activation of these T cell populations, which significantly impacts disease progression^1^. It has recently been demonstrated that CD8^+^ T cells contribute to the state of systemic inflammation and also aggravate liver disease severity in patients with various underlying etiologies of liver disease^8,13–20^. Moreover, not only are immune cells dysregulated in patients with liver cirrhosis, but the soluble immune system is also affected, which can result in systemic hyperinflammation and, subsequently, acute-on-chronic liver failure (ACLF)^21–23^. Certainly, recent evidence indicates that multiple pro-inflammatory markers contribute to the development of liver cirrhosis and its associated complications, which often result in high mortality rates among these patients^22,24,25^.

In this study, we provide a comprehensive characterization of CD4^+^ and CD8^+^ T cells from 72 patients with chronic hepatitis virus infection and alcohol-related liver disease (ARLD) across different stages to decipher immune compartment changes during the course of liver disease. Additionally, we analyzed the soluble immune milieu in patients with decompensated liver cirrhosis.

## Results

### Clinical characteristics of patients with liver disease at different stages

Overall, 72 patients across different stages of liver disease have been analyzed using flow cytometry and data were compared to 24 healthy controls.

In more detail, patients were stratified into different liver disease severity stages: (A) Patients with chronic hepatitis B/D or C virus infection or ARLD, and absence of liver cirrhosis (NLC) (*n* = 12); (B) Patients with chronic hepatitis B/D or C virus infection or ARLD, and evidence of compensated liver cirrhosis (CLC) as defined by clinical, radiological, or histological findings (*n* = 18); (C) Patients with decompensated liver cirrhosis (DLC) as diagnosed by the occurrence of ascites on the basis of chronic hepatitis B/D or C virus infection, ARLD, and/or cryptogenic liver cirrhosis (*n* = 42). Moreover, five of the patients with ascites decompensation presented with ACLF. Bacterial infection of the ascites fluid was absent in all patients included in this study. It is noteworthy that some of the patients included in this study cohort were previously analyzed for other purposes with a focus on other cell subsets and other tissue origins in recently published studies^20,26^. Additionally, peripheral blood from healthy controls (HC) (*n* = 24) were analyzed.

As expected, we observed elevated serum levels of bilirubin, internalized normalized ratio (INR) and decreased albumin values in more severe liver disease (Table S1). Furthermore, in line with previous research^25^we detected an increase in c-reactive protein (CRP) levels with progression of liver disease (Table S1), underscoring the link between systemic inflammation and liver disease severity.

### Frequency and differentiation status of T cells in different stages of liver disease

**Fig. 1:**
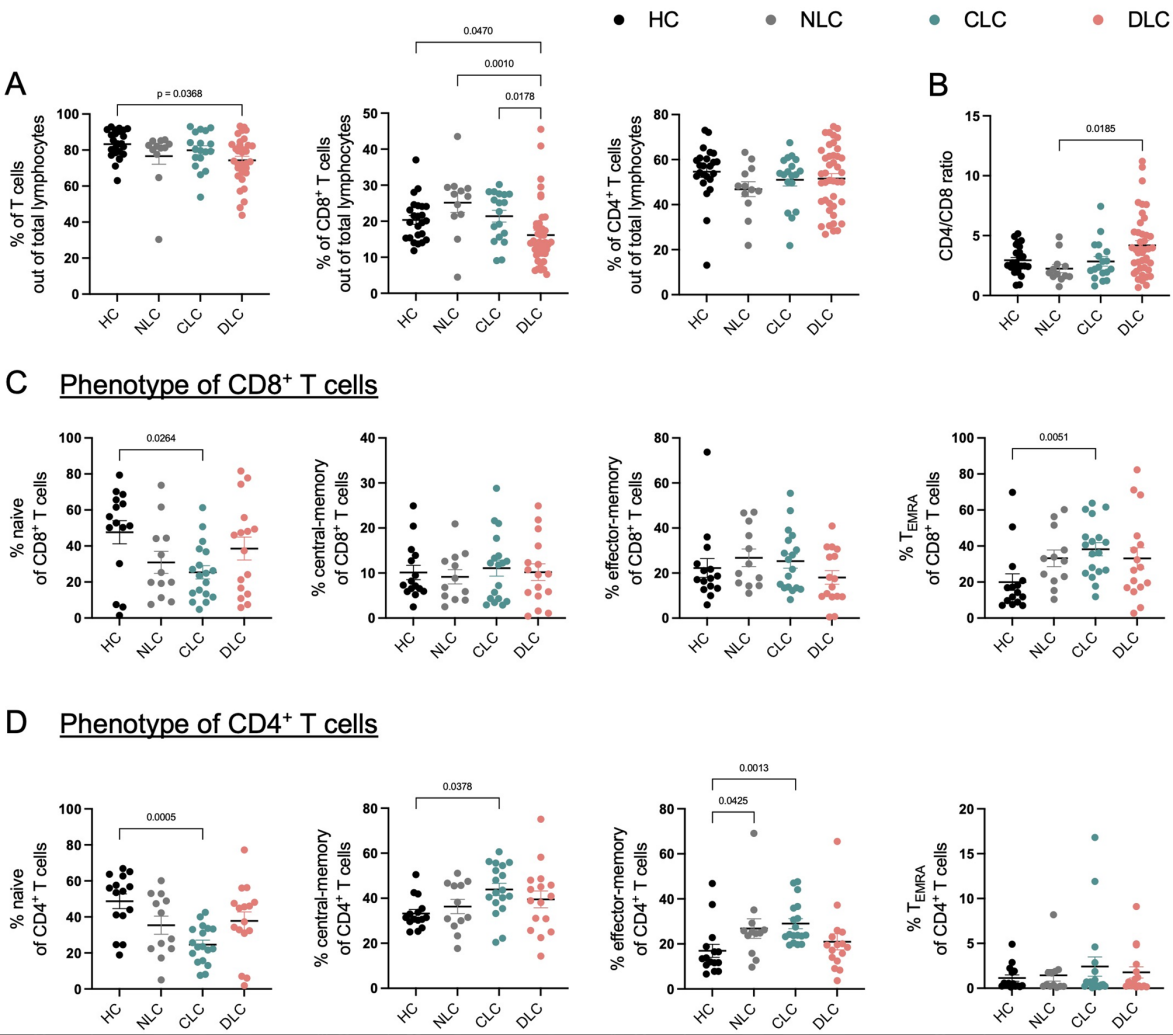
Frequency and differentiation status of T cells in different stages of liver disease. (**A**, **B**) Frequency of total T cells, CD8^+^ T cells, and CD4^+^ T cells out of total lymphocytes (**A**) as well as the CD4/CD8 T cell ratio (**B**) in the peripheral blood of patients with decompensated liver cirrhosis (DLC, *n* = 42), compared to patients with compensated liver cirrhosis (CLC, *n* = 18), patients with chronic viral hepatitis infection and/or ARLD, with the absence of liver cirrhosis (NLC, *n* = 12) and healthy controls (HC, *n* = 24). (**C**, **D**) Differentiation status, revealed by co-staining of CCR7 and CD45RA of CD8^+^ T cells and CD4^+^ T cells, in the same patient cohorts as described in (**A**) and (**B**) (DLC: *n* = 16; CLC: *n* = 18; NLC: *n* = 12; HC: *n* = 15). Kruskal-Wallis test was performed for multiple comparison.

Having established a sizeable patient cohort for downstream immunological analysis, we next analyzed the frequencies of T cells in different stages of liver disease severity. The gating strategy for identification of CD4^+^ and CD8^+^ T cells is depicted in Supplementary Fig. 1. As previously described^20^a gradual decline in total T cell frequencies with more severe liver disease was observed, being significantly diminished in patients with decompensated liver cirrhosis compared to healthy controls (Fig. 1A). This reduction was due to decreased frequencies of CD8^+^ T cells, whereas frequencies of CD4^+^ T cells were relatively stable with worsening liver disease (Fig. 1A). This was further corroborated by an increased CD4/CD8 T cell ratio in patients with decompensated liver cirrhosis compared to patients without liver cirrhosis (Fig. 1B). A subsequent subgroup analysis of patients stratified based on the underlying etiology of liver disease revealed no significant differences between ARLD and hepatitis virus infection related liver disease within the same severity stages (Supp. Fig. 2A).

**Fig. 2:**
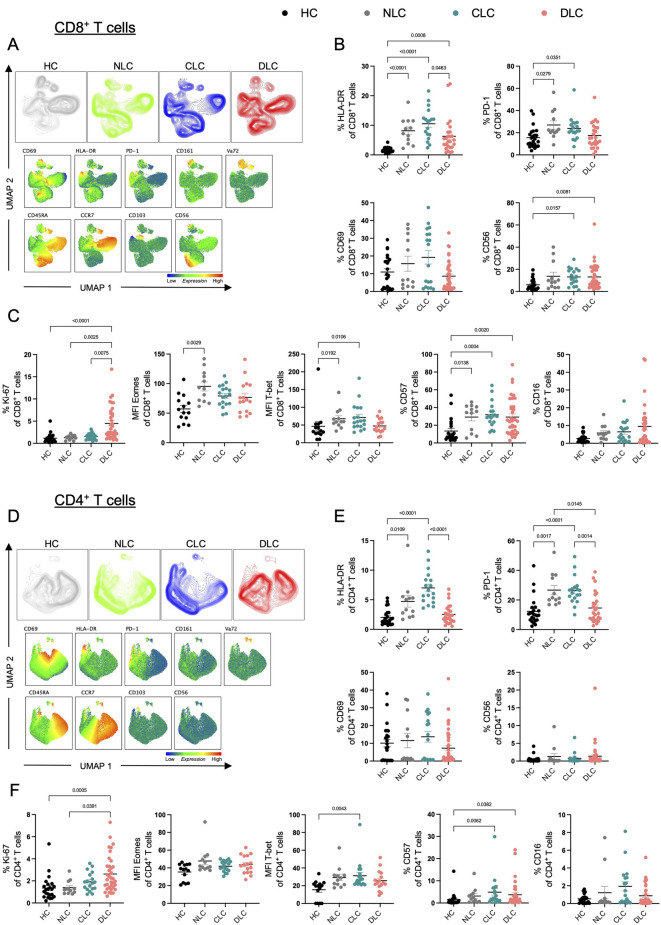
Expression of activation and exhaustion markers in patients across stages of liver disease. (**A**, **D**) UMAP plots displaying the four different patient cohorts (healthy controls (HC), hepatitis virus infection without liver cirrhosis (NLC), compensated liver cirrhosis (CLC), decompensated liver cirrhosis (DLC)) of CD8^+^ (**A**) and CD4^+^ (**D**) T cells, as well as the expression of indicated markers. Per group 10 patients with each 3000 CD8^+^ (**A**) or CD4^+^ (**D**) T cells were exported, concatenated and analyzed using the publicly available UMAP plugin. (**B**, **C**, **E**, **F**) Manual gating on phenotypic markers on CD8^+^ (**B**, **C**) and CD4^+^ (**E**, **F**) T cells from patients across different stages of liver disease compared with healthy individuals as described above (HC: *n* = 14–24; NLC: *n* = 12; CLC: *n* = 18; DLC: *n* = 16–42). Kruskal-Wallis test was performed for multiple comparison.

In addition, we investigated the differentiation status of CD8^+^ and CD4^+^ T cells across different liver disease stages (Fig. 1C, D). In more detail, for CD8^+^ T cells we observed a gradual shift from naive cells in healthy controls to a more pronounced terminally differentiated phenotype in patients with compensated liver cirrhosis (Fig. 1C). In line with this, frequencies of naive CD4^+^ T cells were also found to be higher in healthy controls compared to those with compensated liver cirrhosis, but not decompensated liver cirrhosis (Fig. 1D). Moreover, CD4^+^ T cells from patients with compensated liver cirrhosis displayed a differentiation trajectory towards an effector-memory phenotype, whereas frequencies of effector-memory cells in decompensated cirrhosis were comparable to healthy and/or non-cirrhosis patients (Fig. 1D).

Taken together, CD8^+^ T cells, but not CD4^+^ T cells are decreased in patients with decompensated liver cirrhosis and CD4^+^ as well as CD8^+^ T cells are skewed towards a more differentiated phenotype in compensated, but not decompensated liver cirrhosis.

### Expression of activation and exhaustion markers in patients across stages of liver disease

Having observed a decrease in CD8^+^ T cell frequencies and a shift of CD8^+^ and CD4^+^ T cells towards effector cells in patients with compensated but not decompensated liver cirrhosis, we next wanted to investigate the phenotype of CD8^+^ and CD4^+^ T cells in more detail.

Therefore, we performed high-dimensional data analysis of CD8^+^ and CD4^+^ by applying Uniform Manifold Approximation and Projection (UMAP) analysis (Fig. 2A, D). A distinct clustering of cells co-expressing CD161 and TCRVa7.2, indicative of MAIT cells, was observed in CD8^+^ T cells and to a lesser extent in CD4^+^ T cells (Fig. 2A, D).

Moreover, we observed minor changes in clustering of CD8^+^ T cells and CD4^+^ T cells between patients without liver cirrhosis compared with compensated cirrhosis and decompensated cirrhosis (Fig. 2A, D). In line with the observed increase in the frequency of differentiated effector CD8^+^ and CD4^+^ T cells in patients with compensated liver cirrhosis, we noted a significant elevation in the expression of the activation and exhaustion markers HLA-DR and PD-1 in patients with liver disease, with the highest levels observed in patients with compensated liver cirrhosis (Fig. 2B, E). Remarkably, in patients with decompensated liver cirrhosis, expression of HLA-DR and PD-1 were downregulated compared with compensated cirrhosis (Fig. 2A, E). In addition, expression of CD56 and CD57 were upregulated on CD8^+^ and to a lesser extent CD4^+^ T cells in patients with compensated and decompensated liver cirrhosis compared with healthy controls (Fig. 2B, C, E, F), indicative of higher activation and senescence with advanced liver disease severity. Furthermore, the proliferative capacity, indicated by Ki-67 expression, was significantly elevated in patients with decompensated liver cirrhosis in both CD8^+^ and CD4^+^ T cells (Fig. 2C, F). Moreover, the transcription factor T-bet was elevated in CD8^+^ and CD4^+^ T cells from patients with compensated liver cirrhosis compared with healthy controls, and Eomes was slightly upregulated in CD8^+^ T cells but not CD4^+^ T cells from liver disease patients with absence of cirrhosis (Fig. 2C, F).

With regard to the imbalance in etiologies between subject groups, patients with the same liver disease severity stages were divided into ARLD and hepatitis virus-related liver disease, and the phenotypic changes within the same etiology were compared, as well as the different etiologies with each other (Supp. Fig. 2B, C). Only minor phenotypic differences were observed between ARLD and hepatitis virus-related liver disease from the same disease stage (Supp. Fig. 2B, C). Consistent with the activated and exhausted phenotype observed in compensated but not in decompensated cirrhosis, a significant decrease in the activation marker CD69 on CD8^+^ T cells was observed in patients with decompensated cirrhosis compared to compensated cirrhosis within the group of ARLD patients. In addition, a decline in the senescence marker CD57 was observed in patients with hepatitis virus-related decompensated cirrhosis compared with compensated cirrhosis (Supp. Fig. 2B, C). In line with this, the downregulation of HLA-DR on CD4^+^ T cells in decompensated cirrhosis compared with compensated cirrhosis holds true irrespective of the underlying etiology (Supp. Fig. 2C).

Taken together, with advanced liver disease, CD8^+^ and CD4^+^ T cells shift towards an activated and exhausted phenotype in compensated liver cirrhosis, whereas in the decompensated state, a loss of these markers is present.

### Functional analysis of Circulating T cells across stages of liver disease

Next, we aimed to investigate the functional properties of CD8^+^ and CD4^+^ T cells in more detail to understand the functional consequences of these observations. Therefore, we analyzed the expression of pro-inflammatory and cytotoxic molecules in patients with compensated and decompensated liver cirrhosis compared with healthy controls in unstimulated conditions as well as after 24 h-stimulation with the third-signal cytokines IL-12 + IL-18.

**Fig. 3:**
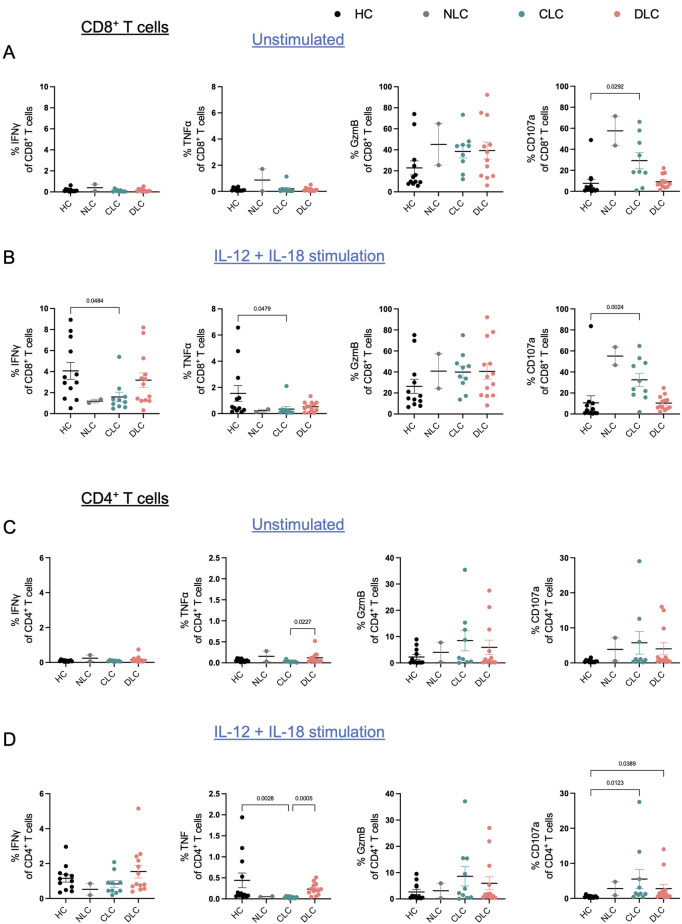
Functional analysis of circulating T cells across stages of liver disease. (**A**–**D**) Expression of pro-inflammatory cytokines and cytotoxic molecules in CD8^+^ (**A**, **B**) and CD4^+^ (**C**, **D**) T cells from patients with viral hepatitis-related liver disease and/or ARLD without liver cirrhosis (NLC, *n* = 2), compensated liver cirrhosis (CLC; *n* = 12), and decompensated liver cirrhosis (DLC, *n* = 12–13) compared with healthy controls (HC, *n* = 12) in unstimulated condition (**A**, **C**) and after IL-12 + IL-18 stimulation for 24 h (**B**, **D**). Functional readouts were compared using Kruskal-Wallis test.

Of note, baseline expression of IFNγ and TNFα appeared to be similar in CD8^+^ T cells of patients across all stages of liver disease compared with healthy controls. With regard to CD4^+^ T cells, TNFα expression in decompensated cirrhosis patients was marginally elevated in comparison with compensated patients (Fig. 3A, C). Furthermore, following stimulation with IL-12 + IL-18, the expression of IFNγ and TNFα was reduced in CD8^+^ T cells from compensated liver cirrhosis in comparison with healthy controls. In contrast, higher CD107 expression was observed in these patients (Fig. 3B). A similar trend was observed for CD4^+^ T cells, where TNFα expression was downregulated after stimulation with IL-12 + IL-18 in compensated cirrhosis compared to healthy controls (Fig. 3D). Of note, levels of Granzyme B expression remained equal across all stages of liver disease and healthy controls (Fig. 3A–D).

In conclusion, CD8^+^ and, to a lesser extent, CD4^+^ T cells from patients with compensated liver cirrhosis show suppressed responsiveness to stimulation with IL-12 + IL-18 compared with healthy individuals.

### Cytokine milieu in patients with decompensated liver cirrhosis

**Fig. 4:**
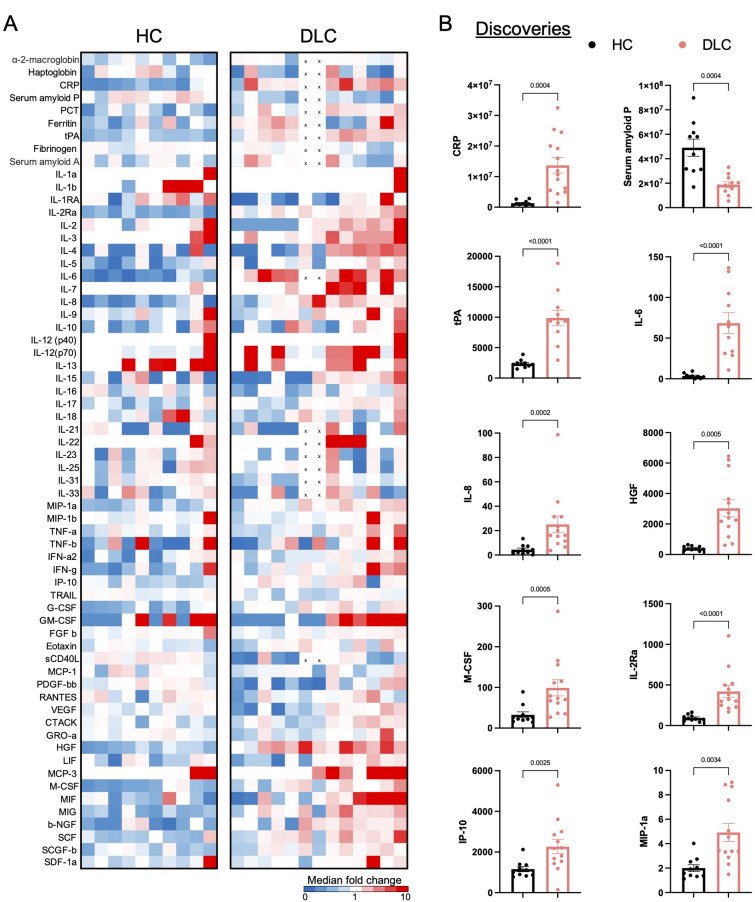
Cytokine milieu in patients with decompensated liver cirrhosis. (**A**) Heatmap of 61 soluble markers, including cytokines, growth factors, chemokines and acute phase proteins in patients with decompensated liver cirrhosis (*n* = 11–13) compared with healthy controls (*n* = 10). Colors indicate median fold change. (**B**) Detected significant discoveries for comparing the soluble markers in patients with decompensated liver cirrhosis with healthy controls. Multiple Mann-Whitney tests were performed for multiple comparisons and two-stage step-up method of Benjamini, Krieger and Yekutieli were performed as a false discovery rate approach.

As it has recently become increasingly evident that systemic inflammation represents a primary contributor to end-stage liver disease^21,27,28^we proceeded to examine the cytokine milieu present in the blood of patients with decompensated liver cirrhosis in comparison with healthy controls. Therefore, we measured 61 soluble factors, including cytokines, chemokines, growth factors and acute phase proteins in the plasma of patients with decompensated liver cirrhosis and healthy controls (Fig. 4A, B). Indeed, elevated levels of several pro-inflammatory cytokines and acute phase proteins were observed in patients with decompensated liver cirrhosis compared with healthy controls (Fig. 4A). In more detail, levels of the pro-inflammatory mediators CRP, tPA, IL-6, IL-8, HGF, M-CSF, IL-2Ra, IP-10, and MIP-1a were significantly increased in patients with decompensated liver cirrhosis compared with healthy controls (Fig. 4B). Interestingly, serum amyloid p, a component of the humoral arm of innate immunity and regulation of tissue remodeling^29^was markedly diminished in patients with decompensated liver cirrhosis (Fig. 4B). Of note, correlation with clinical parameters revealed that within the group of patients with decompensated liver cirrhosis, serum amyloid p as well as the frequency of total T cells, were among the few markers that were significantly negatively correlated with the MELD score (Supp. Fig. 3).

Taken together, the peripheral blood of patients with decompensated liver cirrhosis contains an inflammatory cytokine milieu that is congruent with the observed dysfunctional T cell compartment.

## Discussion

This study provides a comprehensive analysis of circulating CD8^+^ and CD4^+^ T cells in patients across different stages of liver disease and unravels the soluble immune milieu present in the peripheral blood of patients with decompensated liver cirrhosis.

In more detail, we observed that CD8^+^ but not CD4^+^ T cells were diminished in patients with decompensated liver cirrhosis further resulting in an increased CD4/CD8 T cell ratio. Moreover, CD4^+^ and CD8^+^ T cells exhibited a shift towards a more differentiated phenotype in compensated, but not decompensated liver cirrhosis. In addition, with increasing liver disease severity and onset of cirrhosis, both CD8^+^ and CD4^+^ T cells showed a gradual incline of activation and exhaustion marker expression. This is in line with previous observations that describe the cellular immune dysfunction as being dynamic and progressive in parallel to liver disease severity^19,25,30^. Consistent with this, previously published studies emphasize that the clinical course of liver cirrhosis encompasses several disease states and highlight liver cirrhosis as a gradual process^31^. Furthermore, in alignment with the activated and exhausted phenotype of CD8^+^ and CD4^+^ T cells in patients with compensated liver cirrhosis, we observed that both CD8^+^ and CD4^+^ T cells in these patients exhibited diminished responsiveness to cytokine stimulation, consequently resulting in impaired production of pro-inflammatory cytokines and cytotoxic molecules. This further underlines the dysfunctional phenotype of CD8^+^ and CD4^+^ T cells in patients with compensated liver cirrhosis. These results are consistent with those of recent studies that have demonstrated that patients with alcohol-related cirrhosis exhibit profound immune dysregulation, including depletion and functional impairment of MAIT cells^32^as well as increased levels of soluble immune checkpoints such as sTIM-3^33^.

Interestingly, in this study, we observed that patients suffering from decompensated liver cirrhosis exhibited a decline in activation and exhaustion markers, HLA-DR and PD-1, on CD8^+^ and CD4^+^ T cells when compared to patients with compensated cirrhosis. This could be indicative of either a transition from one clinical phase of liver cirrhosis to another^31^and therefore a change from a high-grade inflammatory phenotype towards a low-grade inflammatory phenotype, or, alternatively, reflect the extended exhaustion of the adaptive immune system in these patients. One may also speculate that in patients with decompensated liver cirrhosis, activated effector cells migrate to other tissues or infection sites, resulting in a reduction of activated T cells in the peripheral blood^20,26,34^. This may further explain the enhanced proliferation of CD8^+^ and CD4^+^ T cells observed in patients with decompensated liver cirrhosis, as a replenishment of cells, that have migrated to tissue sites, is necessary. Not only was the T cell compartment in patients with liver cirrhosis prone to activation and exhaustion, but also the soluble immune milieu in the peripheral blood showed an upregulation of pro-inflammatory cytokines and acute-phase proteins, which together signal for dysregulated immune responses^24^.

Overall, this study is confirmatory to previously published work^8,19^ and has certain limitations. It is important to note that the cytokine analysis did not include patients with compensated liver cirrhosis, nor patients with hepatitis virus-related liver disease or ARLD and absence of liver cirrhosis due to a lack of patient samples. Furthermore, it should be acknowledged that there is an imbalance in etiologies between the different stages of liver disease. However, upon stratification of liver disease stages according to underlying etiologies, no significant phenotypic differences emerged between ARLD and hepatitis virus-related liver disease within the distinct stages of liver disease. This is in line with previous studies, that demonstrated that the severity of liver disease and cirrhosis has a more pronounced effect on T cells than the underlying etiology^8,19,35^.

In conclusion, this study demonstrates that, in compensated and, to a lesser extent, decompensated cirrhosis, CD4^+^ and CD8^+^ T cells are skewed towards an activated and dysfunctional phenotype. This further indicates an association between phenotypic and functional changes in the T cell compartment and the different stages of liver disease.

## Materials and methods

### Patient cohort and sample collection

In total, 72 patients with manifest liver disease were enrolled in this study and divided into subgroups: (A) Patients with chronic hepatitis B/D or C virus infection or ARLD and absence of liver cirrhosis (NLC); (B) Patients with chronic hepatitis B/D or C virus infection or ARLD and evidence of compensated liver cirrhosis (CLC); (C) Patients with decompensated liver cirrhosis (DLC) as diagnosed by the occurrence of ascites of either chronic hepatitis B/D or C virus infection, ARLD, and/or cryptogenic liver cirrhosis. All patients were seen at the Department of Gastroenterology, Hepatology, Infectious Diseases and Endocrinology at Hannover Medical School. The diagnosis of cirrhosis was based on clinical, radiological, or histological findings. The existence of ACLF was diagnosed according to the European Association for the Study of the Liver - Chronic Liver Failure (EASL-CLIF) Consortium criteria^28,36,37^. All patients with decompensated liver cirrhosis due to ascites were recruited in the prospective registry INFEKTA (DRKS00010664) and only patients without the presence of SBP, which was diagnosed in patients with ≥ 250 polymorphonuclear cells/mm^3^ ascites fluid^38^were considered for inclusion.

Peripheral blood from patients with liver disease was obtained during daily routine blood sampling, and subsequently PBMCs were isolated using Ficoll density gradient centrifugation followed by cryopreservation in liquid nitrogen according to standard operating procedures. Plasma from decompensated cirrhosis patients was collected from EDTA blood via centrifugation, as previously described, and stored at -80 °C^26^.

Exclusion criteria for this study were hepatocellular carcinoma and/or HIV infection. In addition, peripheral blood from healthy volunteers was collected as controls. Detailed patient characteristics are presented in Tables S1 and S2. Moreover, some of the patients included in this study were previously analyzed for other purposes with a focus on other cell types and other tissue origins in recently published studies^20,26,39^.

### Flow cytometry

Cryopreserved PBMCs were thawed, cells were stained with fluorochrome-labeled monoclonal antibodies and measured by flow cytometry as previously described^26^. For dead cell exclusion, all samples were stained with Fixable Viability Stain 700. For fixation, eBioscience Fixative (FOXP3/Transcription Buffer Staining Set, eBioscience) was used. All antibodies used are listed in Table S3. Samples were acquired on a 16-color LSR Fortessa flow cytometer (BD Biosciences). The obtained data were analyzed by conventional flow cytometry using FlowJo software v10.5.3 (BD Biosciences). For high-dimensional analysis, Uniform Manifold Approximation and Projection (UMAP) were performed using the open-access available FlowJo plugin^40^. CD8^+^ as well as CD4^+^ T cells were identified as single, alive CD14^−^CD19^−^CD3^+^ cells expressing CD8 or CD4 (Supp. Fig. 1A).

### Functional assays

PBMCs were either stimulated with IL-12 + IL-18 for 24 h as previously described^39^or left untreated. Briefly, cryopreserved PBMCs were thawed, followed by stimulation with IL-12 (10 ng/mL; Miltenyi Biotech) + IL‐18 (100 ng/mL; MBL International Corporation) to induce cytokine response. Additionally, in selected experiments stimulation with PMA/ionomycin (2 µg/mL; 500 ng/mL) was performed as a positive control. For the last 6 h of stimulation, Brefeldin A (2 µg/mL; Golgi Plug; BD Biosciences), Monensin (Golgi Stop; BD Biosciences) and CD107a were added to assess degranulation. Subsequent to the incubation period, cells were stained both intra- and extracellularly with fluorochrome-labeled monoclonal antibodies, and analyzed by flow cytometry as previously described^26^.

### Soluble immune marker measurement

In total, 61 soluble markers, including cytokines, growth factors, chemokines and acute phase proteins were measured in the plasma from patients with decompensated liver cirrhosis and healthy controls using the Luminex-based multiplex bead assays (Human Cytokine Screening Panel, 12007283; Human Cytokine 17-plex Assay, M5000031YV; Human Acute Phase 4-plex Panel, 171A4C09M; all Bio‐Rad Laboratories, Hercules, CA) according to the manufacturer’s instructions and optimized protocols. All samples were analyzed using BioPlex Manager 6.0 software. Cytokine values that were below the detection range were determined as the lowest possible calculated concentration divided by two.

### Statistical analysis

Statistical analyses were performed using GraphPad Prism software 10.4 (GraphPad Software, San Diego, CA, USA). Firstly, the distribution of datasets was assessed using the D’ Agostino & Pearson normality test. For the comparison of two unmatched and not normally distributed datasets the Mann-Whitney test was used. For all multiple comparisons, non-parametric tests were applied because of the small sample sizes. Therefore, the Kruskal-Wallis test was performed. Correlations between non-parametric datasets were analyzed using Spearman’s r coefficients. Details regarding statistical tests are displayed in the figure legends, and significances are indicated as actual p-values in the graphs.

### Study approval

Written informed consent was obtained from all participants before inclusion. The study was approved by the Ethics Committee of Hannover Medical School (3188-2016), and the study protocol conforms to the ethical guidelines of the 1975 Declaration of Helsinki.

## Supplementary Information

Below is the link to the electronic supplementary material.

Supplementary Material 1

## Data Availability

The datasets used and/or analyzed during the current study are available from the corresponding author on reasonable request.
