# Supplementary material for: T cell dysregulation reflects disease stage in hepatitis virus and alcohol-related liver disease
Source: Sci Rep. 2025 Sep 30;15:34108. doi: 10.1038/s41598-025-18624-4 (PMC12485000; doi:10.1038/s41598-025-18624-4)
Supplement: Supplementary file 1 — Supplementary Material 1 [file 41598_2025_18624_MOESM1_ESM.pdf]

Supplemental material

T cell dysregulation reflects disease stage in hepatitis virus and alcohol-related liver disease

Christian Niehaus, Yin-Han Chou, Helena Lickei, Ayesha Lietzau, Roni Souleiman, Benjamin Maasoumy, Heiner Wedemeyer, Christine S. Falk, Anke RM Kraft, Markus Cornberg

Table of contents

Supplementary Table S1

Supplementary Table S2

Supplementary Table S3

Supplementary figure legends

Supplementary Figure 1

Supplementary Figure 2

Supplementary Figure 3

Supplementary Table S1. Patient characteristics of all patients included in this study.

Parameter	Healthy	No cirrhosis	Compensated cirrhosis	Decompensated cirrhosis	
**Number of patients**	24	12	18	Total: 42	
				No ACLF	ACLF
**Sex**					
- Female	12	8	11	15	1
- Male	12	4	7	27	4
**Age**	58.1 ±7.8	53.3 ±10.6	54.8 ±13.6	63.4 ±8.9	67 ±6.6
**CHILD-Pugh Score (A/B/C)**	/	/	18/0/0	0/29/13	0/0/5
**MELD score**	/	/	8 ±2	14 ±8	21 ±13
**ACLF (grade 1/2/3)**	/	/	/	/	5/0/0
**AST (U/L)**	/	72 ±43.6	64.5 ±52.2	53.3 ±29.1	50.4 ±32.9
**ALT (U/L)**	/	109.3 ±87.8	75.1 ±105.8	28.8 ±24.1	25 ±16.6
**Bilirubin (umol/L)**	/	11.9 ±5.6	13.1 ±6.4	42.1 ±49.9	78.8 ±72.7
**Albumin (g/dL)**	/	4.2 ±0.5	4.1 ±0.6	2.8 ±0.5	2.6 ±0.9
**INR**	/	1.1 ±0.1	1.2 ±0.1	1.4 ±0.4	1.7 ±0.7
**CRP (mg/L)**	/	1.05 ±0.4	4.0 ±3.4	21.4 ±17.9	20.9 ±16.4
**Etiology**					
- HCV	/	5	6	5	1
- HBV/HDV	/	5	8	4	0
- ARLD	/	2	4	30	4
- Cryptogenic	/	/	/	3	0

All values provided as mean ± SD
ACLF, acute-on-chronic liver failure; AKI, acute kidney injury; ALT, alanine transaminase; ARLD, alcohol-related liver disease; AST, aspartate transaminase; CRP, C-reactive protein; HE, hepatic encephalopathy; INR, International Normalized Ratio; MELD, model of end stage liver disease.

Supplementary Table S2. Extended clinical data on patients with hepatitis virus-related liver disease.

Etiology	HBsAg (IU/mL)	HBV-DNA (IU/mL)	HCV-DNA (IU/mL)	HDV-RNA (IU/mL)	Antiviral therapy at the timepoint of sample collection (y/n)
**HCV (n=17)**	-	-	5.75E+05 ±774751	-	0/17
**HBV/HDV (n=17)**	14801 ±7617	All neg.	-	7.36E+05 ±903595	10/7

All values provided as mean ± SD; HBsAg, hepatitis B surface antigen.

Supplementary Table S3. Antibodies used for flow cytometry.

Antibody name	Color	Clone	Supplier
anti-CD45RA	FITC	HI100	BioLegend
anti-Ki-67	Alexa Fluor 488	Ki-67	BioLegend
anti-CD107a (LAMP-1)	Alexa Fluor 647	H4A3	BioLegend
anti-CD45 (LCA)	Alexa Fluor 700	HI30	BioLegend
anti-CD57	APC	QA17A04	BioLegend
anti-CD8	APC-Fire750	RPA-T8	BioLegend
anti-CD279 (PD-1)	Brilliant Violet 421	EH12.2H7	BioLegend
anti-IFN-γ	Brilliant Violet 421	4S.B3	BioLegend
anti-T-bet	Brilliant Violet 421	4B10	BioLegend
anti-CD3	Brilliant Violet 510	UCHT1	BioLegend
anti-CD161	Brilliant Violet 605	HP-3G10	BioLegend
anti-CD16	Brilliant Violet 650	3G8	BioLegend
anti-HLA-DR	Brilliant Violet 650	L243	BioLegend
anti-TNF-α	Brilliant Violet 650	Mab11	BioLegend
anti-CD14	Brilliant Violet 711	M5E2	BioLegend
anti-CD19	Brilliant Violet 711	HIB19	BioLegend
anti-Granzyme B	PE	QA16A02	BioLegend
anti-CD197 (CCR7)	PE	G043H7	BioLegend
anti-CD56	PE-Cy7	QA17A16	BioLegend
anti-CD4	PerCP/Cy5.5	RPA-T4	BioLegend
anti-CD69	PE-Dazzle594	FN50	BioLegend
anti-IL-17A	PE-Dazzle594	BL168	BioLegend
anti-Eomes	PE-eFluor 610	WD1928	ThermoFisher
Fixable Viability Stain 700			BD Biosciences

Supplementary Figure 1. Gating strategy.

In order to identify $CD8^+$ and $CD4^+$ T cells, first a time gate was set to exclude clumps. Next, gating for lymphocytes followed by exclusion of doublet events were performed. Thereafter, removal of dead cells and $CD14^+CD19^+$ cells were conducted and subsequently gating on $CD3^+$ cells were performed to identify T cells. Then, the gating for CD8 and CD4 was performed in order to identify $CD8^+$ T cells and $CD4^+$ T cells, respectively.

Supplementary Figure 2. Stratification of liver disease stages into underlying etiologies.

Patients from the different liver disease (no liver cirrhosis, NLC; compensated liver cirrhosis, CLC; and decompensated liver cirrhosis, DLC) stages were stratified into patients with alcohol-related liver disease (ARLD) (n=40) and hepatitis virus infection-related liver disease (n=34). Shown are (A) frequency of total T cells, $CD8^+$ T cells and $CD4^+$ T cells out of total lymphocytes, respectively. (B, C) Expression of activation, exhaustion, senescence, and proliferation markers as well as transcription factor expression on $CD8^+$ (B) and $CD4^+$ (C) T cells are depicted. For multiple comparison, Kruskal-Wallis test was performed.

Supplementary Figure 3. Correlation matrix of $CD8^+$ and $CD4^+$ T cell frequencies, plasma cytokines and clinical markers in patients with decompensated liver cirrhosis.

Correlation between clinical markers, plasma cytokines, and the frequency of $CD4^+$ and $CD8^+$ T cells in patients with decompensated liver cirrhosis. For correlations between nonparametric datasets Spearman´s r coefficients were calculated and

depicted using a color-scale from blue (r= -1) to red (r= 1). Significances are shown by asterisks in each square. * $p<0.05$; ** $p<0.01$; *** $p<0.001$; **** $p<0.0001$.
